# Delayed Diagnosis of Quadriceps Tendon Rupture

**DOI:** 10.7759/cureus.71270

**Published:** 2024-10-11

**Authors:** Joseph Levinson, Alyssa Mixon

**Affiliations:** 1 Physical Medicine and Rehabilitation, University of Virginia School of Medicine, Charlottesville, USA

**Keywords:** delayed diagnosis, physical and rehabilitation medicine, quadriceps muscle, tendon injuries, treatment outcome

## Abstract

Quadriceps tendon (QT) injury classically presents with pain, inability to actively extend the knee, and a palpable defect in the suprapatellar region. Unilateral eccentric contraction and hyperflexion after ground level fall are the most common mechanisms of injury. Delayed QT repair may require additional operative procedures and is associated with poorer post-operative functional outcomes, persistent knee extensor weakness, and decreased range of motion. This report demonstrates a case of delayed diagnosis of QT rupture after a fall with injury to the contralateral extremity. Initial evaluation and X-ray did not reveal the rupture. Six weeks later, after multiple re-admissions to the acute hospital from inpatient rehabilitation for sepsis, Physical Medicine and Rehabilitation (PM&R) was consulted and noted a QT rupture on exam. The rupture was confirmed on MRI and the patient underwent surgery within days. This case demonstrates the value of an interdisciplinary approach and involvement of PM&R throughout the care continuum.

## Introduction

Quadriceps tendon (QT) injuries make up 1.3% of ligament and tendon injuries [1]. QT injury is more common in males and those over 40 years of age [2]. Injury to the QT typically occurs during eccentric contraction with indirect trauma or hyperflexion with load [1]. While less common, spontaneous rupture may also occur, and is often associated with predisposing comorbidities associated with tendinopathy such as diabetes, thyroid disease, renal disease, elevated lipids or inflammatory disease, or medication exposure such as fluoroquinolone or steroid use [2,3].

Diagnosis of QT rupture is based on history and physical, including the classic clinical triad of acute pain, lack of active extension at the knee, and a palpable suprapatellar gap [1,4]. X-ray and MRI can be helpful in diagnosis and operative planning as well [1]. Ultrasound can be helpful for initial imaging, but MRI is the gold standard [5]. Many partial ruptures can be treated conservatively with therapeutic aspiration of traumatic hematoma or effusion in conjunction with rest, ice, compression, and elevation. A hinged brace can be used, initially locked in extension then opened in a stepwise fashion once a straight leg raise can be performed without pain. At that point, the patient should start isometric quadriceps contraction, straight leg raises and active knee flexion to ninety degrees. Once this can be done without pain, the patient can progress to knee extension exercises, beginning at thirty degrees and progressively increasing the range of motion. Surgery is required for complete rupture [5].

Early detection of QT rupture is vital, as delayed presentation or diagnosis can cause muscle contracture and scarring, which may require additional operative procedures or alternative techniques. If not diagnosed and treated in a timely manner, it may lead to persistent weakness or stiffness of the quadriceps muscle including postoperatively [6]. A systematic review by Ciriello et al. found that delay in surgical repair was the only factor causing poorer outcomes amongst all primary repair techniques [3]. This report presents a case of delayed diagnosis of QT rupture in a patient following several admissions to both an acute hospital and an inpatient rehabilitation facility and the importance of early consultation for Physical Medicine and Rehabilitation (PM&R).

## Case presentation

**Figure 1:**
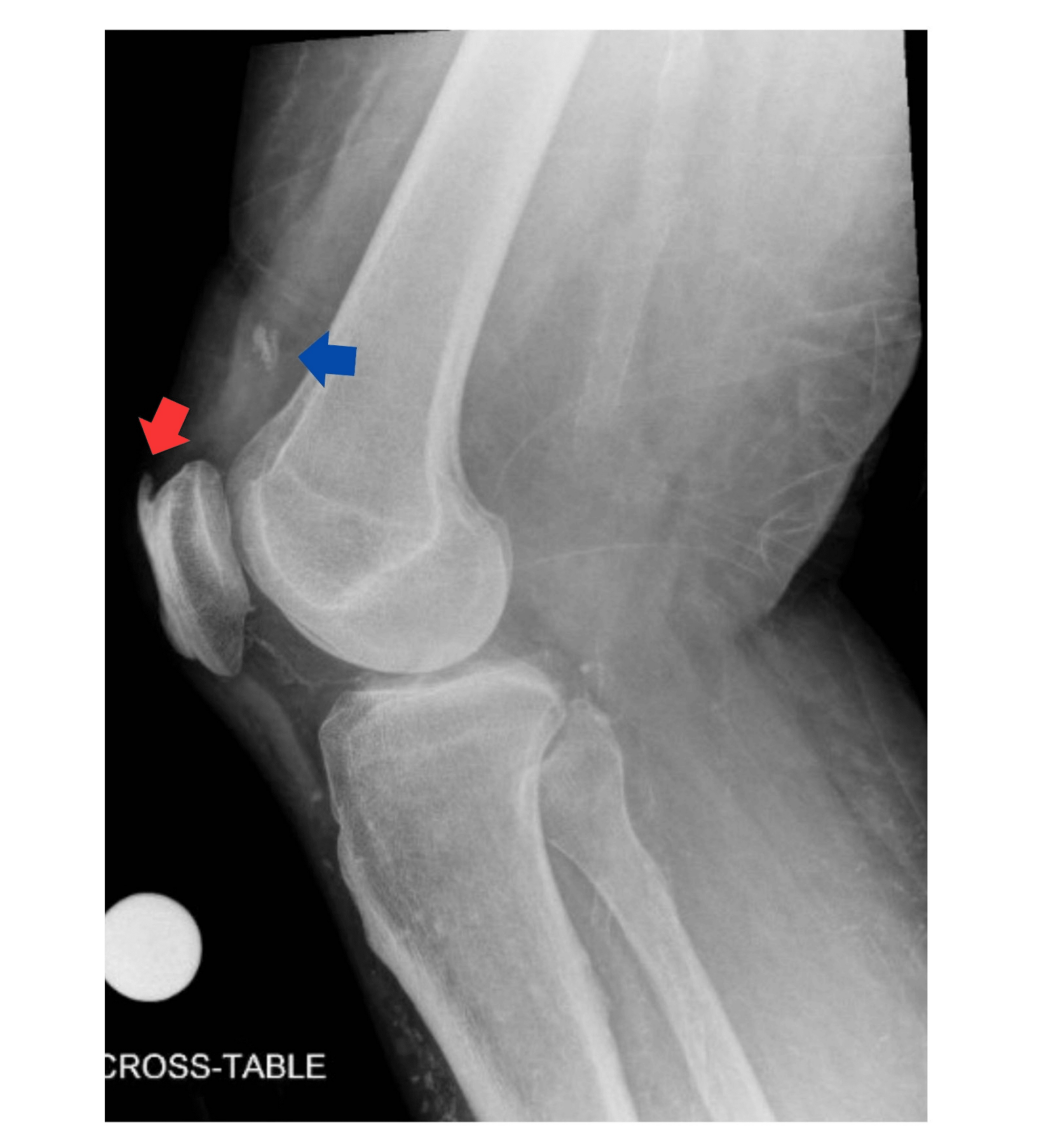
Right Knee X-Ray, Lateral View Lateral view of right knee X-ray taken on initial presentation, demonstrating anterior patellar spur (red arrow) as well as an osseous body overlying the suprapatellar bursa (blue arrow), but no acute fracture.

**Figure 2:**
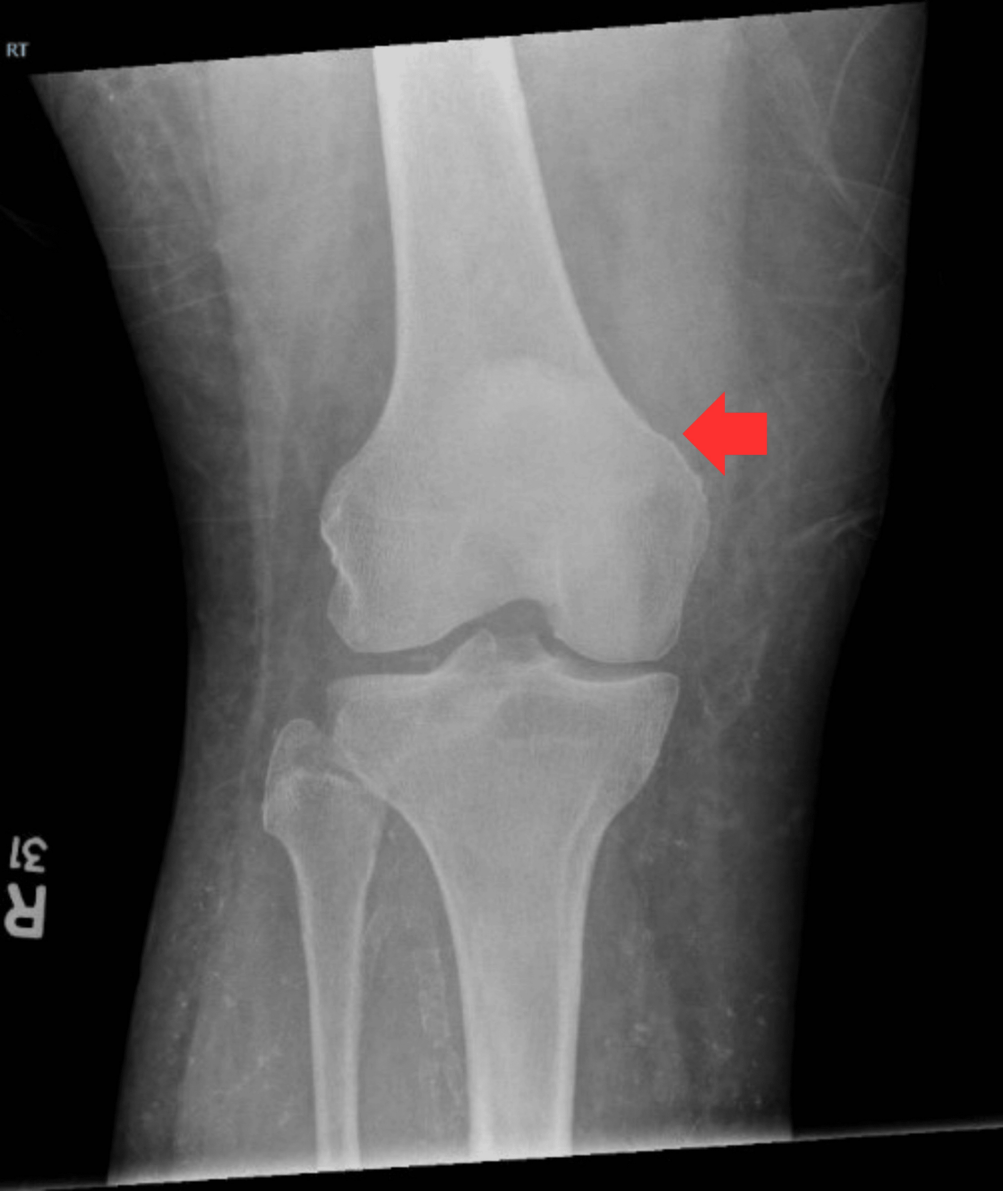
Right Knee X-Ray, Anteroposterior View Anteroposterior view of right knee X-ray taken on initial presentation. There is no abnormality identified in the distal femur or patella on this anteroposterior view. The red arrow points to the patella and distal femur.

A 68-year-old male with an extensive past medical history including type two diabetes mellitus, heart failure with preserved ejection fraction, and history of deep vein thrombosis and pulmonary embolism on chronic anticoagulation, presented after a fall from a stepladder with a left lower extremity laceration. Initial evaluation did not note impaired lower extremity strength or active range of motion. A right knee X-ray was performed due to right knee pain and noted the presence of an anterior patellar spur as well as an osseous body overlying the suprapatellar bursa, but no acute fracture (Figures 1, 2).

The patient underwent debridement and wound vac placement to his left lower extremity twice and was discharged to acute inpatient rehabilitation. Upon initial admission to acute inpatient rehabilitation, manual motor testing of the bilateral lower extremities, in particular bilateral knee extension, was limited by pain. There was no documentation regarding the presence or absence of a suprapatellar gap. Therapy evaluation and repeat physician physical examination continued to be limited by lower extremity pain, as well as significant orthostasis with standing. The patient was sent to the acute care hospital for abdominal pain and tachycardia within several days, prior to completion of formal manual motor testing.

At the acute care hospital, the patient was re-admitted and treated for septic shock due to a urinary tract infection. During physical therapy evaluation the day prior to discharge back to acute inpatient rehabilitation, the therapist noted right knee buckling and visible quadriceps muscle contraction, but no leg movement with right knee extension testing. The patient was discharged back to acute inpatient rehabilitation, but right knee extension range of motion and strength testing were not performed then due to right knee pain. Therapy evaluation was notable for deferral of ambulation due to safety concerns related to right knee buckling. After several days in acute rehabilitation, the patient developed sinus tachycardia, hypotension and shortness of breath requiring another acute care transfer.

**Figure 3:**
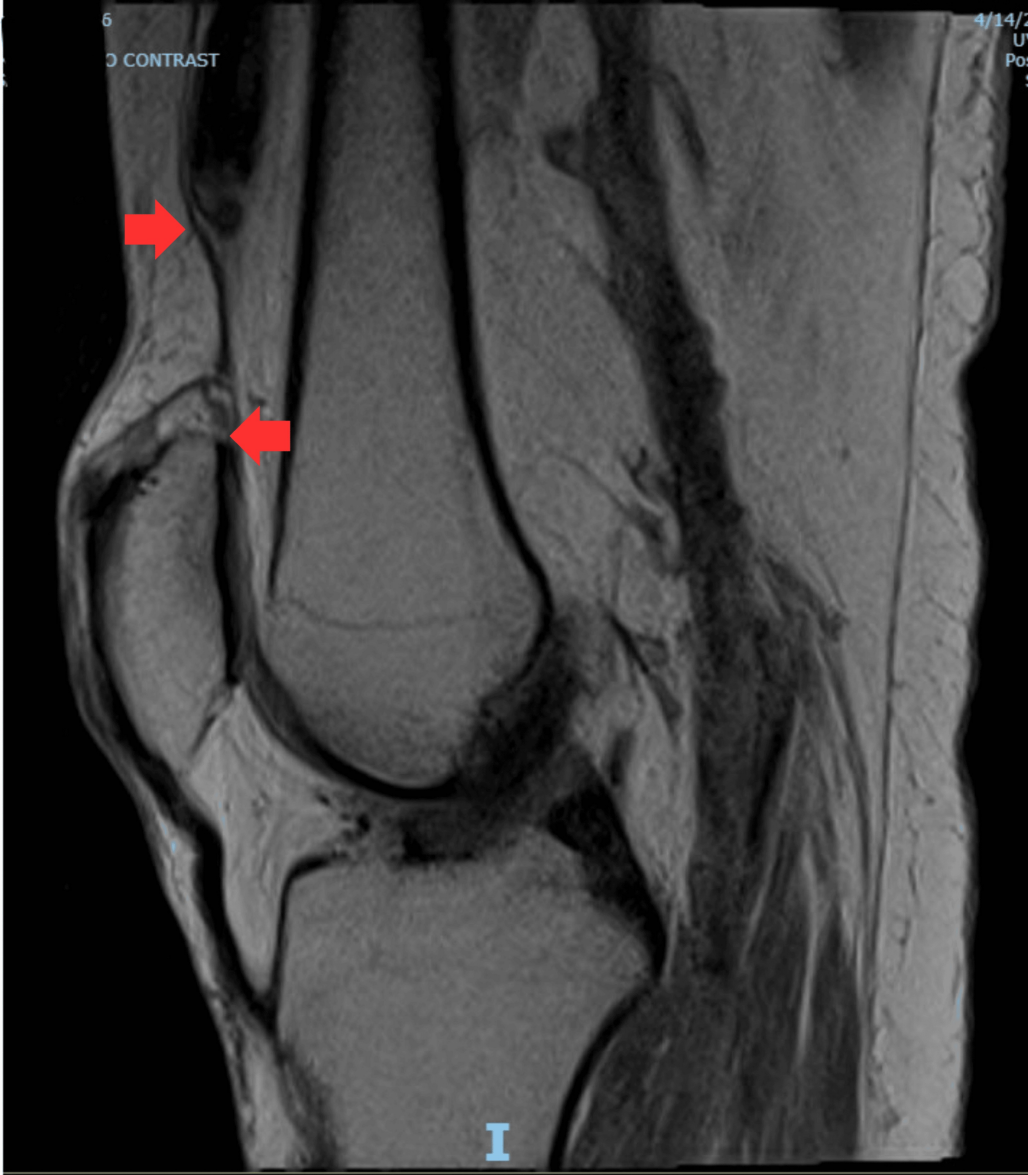
Right Knee MRI, Lateral View Lateral view of right knee MRI (T1) performed at physiatrist’s recommendation demonstrating a “complete tear of the distal quadriceps tendon attachment with 2.7cm of retraction,” felt possibly subacute due to fluid in the region. The red arrows indicate the void left by QT retraction. QT: Quadriceps tendon

**Figure 4:**
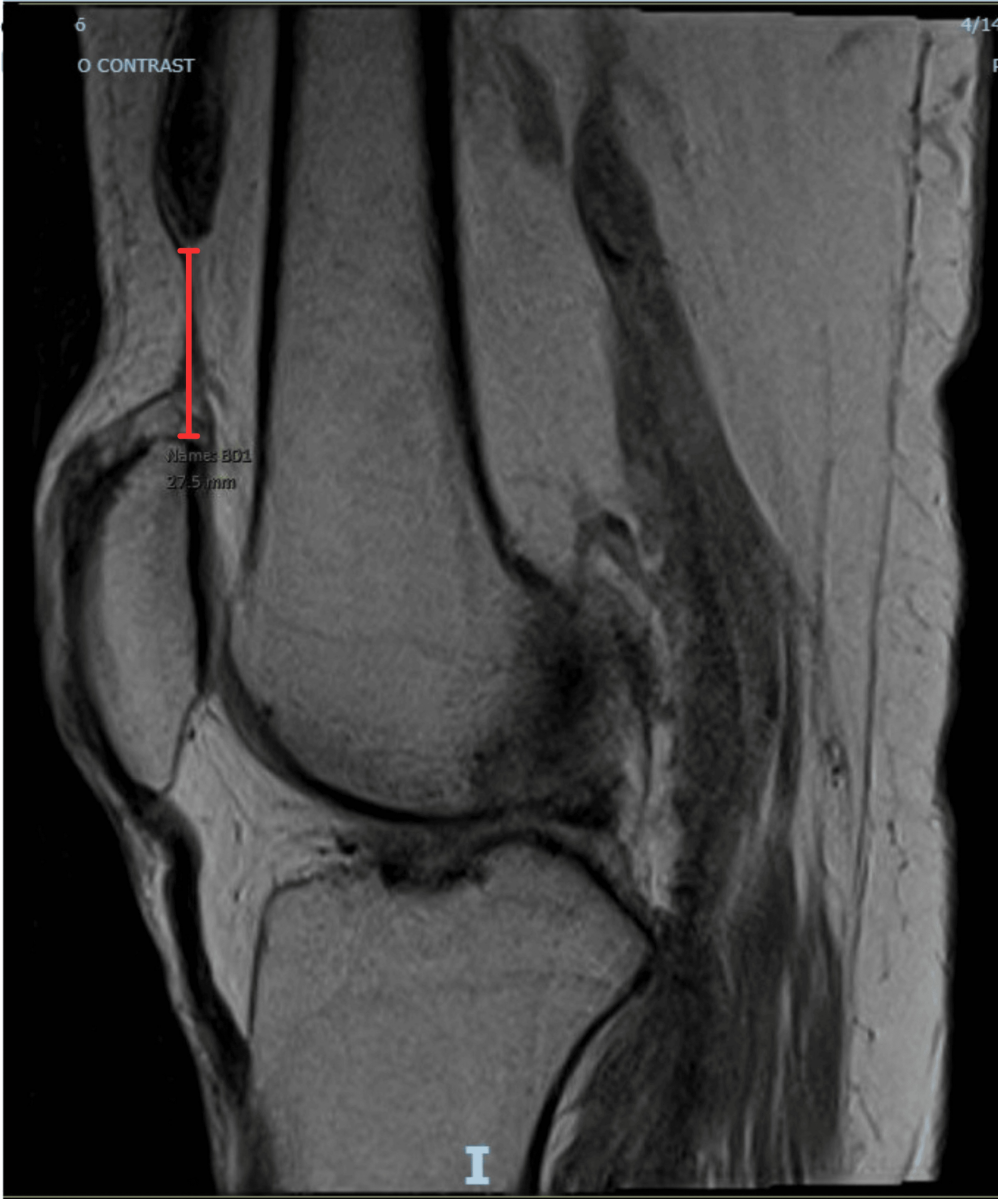
Right Knee MRI, Lateral View with Measurement Lateral view of right knee MRI (T1) again, with extent of retraction as measured by reading radiologist (red line, measuring 2.75 cm).

During the patient’s third hospitalization, he was treated for sepsis attributed to his left lower extremity wound. For this acute care admission, a PM&R consult was placed for evaluation of the patient’s candidacy to return to acute rehabilitation. The primary team noted during their call to the consulting physiatrist that the therapy team was concerned about the patient’s right knee buckling. The consulting physiatrist noted 1/5 strength of right knee extension with inability to fully extend, pain with active knee extension, and a palpable defect at the distal right QT. MRI of the right knee performed at the physiatrist’s recommendation demonstrated a “complete tear of the distal quadriceps tendon attachment with 2.7cm of retraction,” felt possibly subacute due to fluid in the region (Figures 3, 4).

Orthopedic surgery was consulted and also noted a palpable defect over right distal QT with approximately 30-degree extensor lag. Several days later, the patient underwent surgical repair of the right QT. A right V-Y lengthening quadricepsplasty was also performed due to significant tension in the repair.

The patient required maximum assistance from staff both before and after his procedure and was ultimately discharged to a skilled nursing facility (SNF). At his first post-operative visit, he was transitioned from touch down weightbearing in an incremental range of motion (IROM) brace locked in extension, to weightbearing as tolerated with IROM brace locked in extension. The knee brace was liberalized to 0-30 degrees, increasing by 30 degrees every two weeks with plans to follow up in one month.

## Discussion

QT rupture is a clinical diagnosis based on history of acute pain and examination findings with inability to actively extend knee and suprapatellar gap [1,4]. While QT rupture is a clinical diagnosis, X-ray may demonstrate findings such as patella baja, suprapatellar mass, suprapatellar calcific densities, and obliteration of the QT shadow [1]. Often X-ray is sufficient and MRI is not required, although MRI may be used in chronic QT tears for operative planning as well ascertaining the degree of retraction and atrophy [1]. Ultrasound in combination with physical examination was found in one study to be sensitive in QT rupture with a value of 1.0, however specificity was 0.67 and positive predictive value was 0.88. MRI is felt to be the gold standard of QT rupture confirmation, with sensitivity, specificity and positive predictive value 1.0 in the same study [1,7].

In the available literature, the majority of case reports and case series either fail to note the cause of delayed QT rupture repair, or note it was due to delayed patient presentation [3]. One case report does describe an instance of delayed diagnosis of QT rupture in a patient with similar trauma mechanism to our case. In this case an individual fell from a ladder and presented with inability to bear weight on the affected extremity and mechanical symptoms [8]. He was initially diagnosed with anterior cruciate ligament tear based on physical exam and MRI. After failing to improve after two months of therapy, he re-presented to orthopedics and was noted to have a palpable QT defect, incomplete active knee extension, and upon re-review of the MRI a QT rupture was noted. The patient underwent repair, and at ten month follow up had persistent quadriceps weakness with full range of active knee extension.

There are numerous reasons delay in patient presentation or diagnosis of QT rupture may occur. Early diagnosis of QT rupture can be made more difficult by hemarthrosis masking the suprapatellar gap, retention of some active knee extension due to an intact patellar retinaculum, or simply delayed patient presentation [1]. A systematic review of a number of case series examining QT rupture repair found worse or mixed outcomes in the delayed repair group when compared with timely repair [3]. This has been attributed to prolonged quadriceps disuse which often ultimately requires the use of graft, mesh, or alternative surgical techniques to lengthen the muscle, and may result in persistent weakness and/or decreased range of motion at the quadriceps [6]. However, there are case reports of successful outcomes after delayed repair utilizing some of these techniques [9].

Our patient was not misdiagnosed, but rather had a delay in diagnosis. This patient was medically complex, and on initial presentation had a complex wound on the contralateral extremity. Initial evaluation did not include a documented strength or range of motion exam of his lower extremities, though initial examination after acute injury can be limited as noted above. A thorough initial examination after trauma as well as serial examinations become important in these cases.

Initial imaging included X-ray of the right knee with an osseous body overlying the suprapatellar bursa, however the patella was in a normal position (Figures 1, 2). While there may be evidence of QT rupture on X-ray, it is a clinical diagnosis first and foremost. As in our patient, MRI is useful in equivocal cases and for operative planning, particularly in patients with delayed presentation or diagnosis (Figure 3 and Figure 4). Furthermore, the patient’s physical examinations and therapy sessions were often limited by pain and comorbidities, including significant orthostasis and contralateral extremity wound pain. It is also possible this injury occurred at some point in time after his initial fall from a ladder, although this seems the most likely mechanism of injury given lack of other reported fall or trauma. However, spontaneous rupture can occur, although no culprit medications or prior history of tendinopathy were noted.

Prior studies have demonstrated benefit to early acute care PM&R consultation. A Canadian level 1 trauma center started a PM&R consult service and subsequently noted a decrease in length of stay and complication rate among trauma patients. However, this benefit was only significant if PM&R was consulted within the first eight days of admission [10]. The PM&R BOLD Initiative is an effort from the American Academy of Physical Medicine and Rehabilitation (AAPM&R) which aims to delineate the path forward for the specialty of PM&R and its role throughout the care continuum. This initiative notes that physiatry involvement in the acute hospital setting can reduce cost while improving outcome and patient experience as well [11].

## Conclusions

Delay in recognition of QT rupture may alter surgical technique and result in less ideal outcomes post-operatively. Physiatrists are uniquely well trained in identifying injuries that impact functional status. Early diagnosis and treatment of QT rupture may allow for better participation in rehabilitation, with improved functional and medical outcomes. This may have impacted our patient’s ultimate disposition, and allowed him to discharge home, rather than to a SNF. This case emphasizes the importance of a multidisciplinary approach and early involvement of PM&R physicians during the acute hospitalization, in line with the PM&R BOLD Initiative Rehabilitation Care Continuum practice area.
